# Understanding pregnancy as a teachable moment for behaviour change: a comparison of the COM-B and teachable moments models

**DOI:** 10.1080/21642850.2021.2014851

**Published:** 2021-12-30

**Authors:** Lauren Rockliffe, Sarah Peters, Alexander E. P. Heazell, Debbie M. Smith

**Affiliations:** a Faculty of Biology, Medicine and Health, Manchester Centre for Health Psychology, School of Health Sciences, University of Manchester, Manchester, UK; b Faculty of Biology, Medicine and Health, Maternal and Fetal Health Research Centre, School of Medical Sciences, University of Manchester, Manchester, UK; c St. Mary’s Hospital, Central Manchester University Hospitals NHS Foundation Trust, Manchester Academic Health Science Centre, Manchester, UK

**Keywords:** Pregnancy, psychological theory, health behaviour, COM-B, teachable moment

## Abstract

**Objectives:**

Theoretical models have informed the understanding of pregnancy as a ‘teachable moment’ for health behaviour change. However, these models have not been developed specifically for, nor widely tested, in this population. Currently, no pregnancy-specific model of behaviour change exists, which is important given it is a unique yet common health event. This study aimed to assess the extent to which factors influencing antenatal behaviour change are accounted for by the COM-B model and Teachable Moments (TM) model and to identify which model is best used to understand behaviour change during pregnancy.

**Design:**

Theoretical mapping exercise.

**Methods:**

A deductive approach was adopted; nine sub-themes identified in a previous thematic synthesis of 92 studies were mapped to the constructs of the TM and COM-B models. The sub-themes reflected factors influencing antenatal health behaviour.

**Findings:**

All sub-themes mapped to the COM-B model constructs, whereas the TM model failed to incorporate three sub-themes. Missed factors were non-psychological, including practical and environmental factors, social influences, and physical pregnancy symptoms. In contrast to the COM-B model, the TM model provided an enhanced conceptual understanding of pregnancy as a teachable moment for behaviour change, however, neither model accounted for the changeable salience of influencing factors throughout the pregnancy experience.

**Conclusions:**

The TM and COM-B models are both limited when applied within the context of pregnancy. Nevertheless, both models offer valuable insight that should be drawn upon when developing a pregnancy-specific model of behaviour change.

## Introduction

During pregnancy, women often have increased motivation to improve their lifestyle and health behaviours (Lindqvist et al., 2017; O’Brien et al., 2017). For this reason, pregnancy is commonly referred to as a ‘teachable moment’, which has been defined as a ‘naturally occurring life transition […] or health event […] thought to motivate individuals to spontaneously adopt risk-reducing health behaviours’ (McBride, Emmons, & Lipkus, 2003, p. 156).

Teachable moments can present valuable opportunities for professionals to promote healthy behaviours, as patients may be more receptive to health messages at this time (e.g. cessation of cigarette smoking). In particular, pregnancy provides a unique opportunity for health professionals to deliver health promotion, as women have increased frequency of contact with health professionals over the course of their pregnancy (National Institute for Health and Care Excellence, 2021).

Eating behaviour, smoking, alcohol use, and physical activity are four key modifiable behavioural risk factors for adverse pregnancy outcomes and lifelong non-communicable diseases (Hayes et al., 2021; World Health Organisation, 2020). Supporting and encouraging pregnant women to make healthy changes to these behaviours is therefore particularly important, as this can reduce the risk of pregnancy-related conditions such as preeclampsia and gestational diabetes, and obstetric complications such as caesarean birth, miscarriage, and stillbirth (Escañuela Sánchez, Meaney, & O’Donoghue, 2019; Liu et al., 2020; Sundermann et al., 2019; Yang et al., 2019). Furthermore, improving pregnancy-related behaviours (i.e. those that also occur outside of pregnancy) may mean healthy changes are maintained into the postnatal period and beyond, potentially improving long-term health outcomes for both the mother and child, and for future pregnancies.

However, the concept of the ‘teachable moment’ has remained largely untested and poorly theorised (Lawson & Flocke, 2009), particularly in relation to pregnancy. McBride et al. (2003) first posited the psychological characteristics underlying teachable moments within the context of smoking cessation. The authors argue that an effective teachable moment is cued by a health event that (1) increases personal perception of risk and outcome expectations, (2) provokes a strong emotional response, and (3) causes a redefinition of self-concept or social role. The greater the degree to which all the domains are acted upon, the greater the likelihood the teachable moment will result in behaviour change (McBride et al., 2003). It is clear that pregnancy has the potential to act upon each of these psychological domains. For example, it is likely that the experience of being pregnant will prompt the mother to consider the associated risks of the pregnancy for both herself and her unborn child, and outcomes related to her current behaviour (Coll et al., 2017). Pregnancy is also an experience that is likely to provoke a strong emotional response (McLeish & Redshaw, 2017; Nakamura, Sato, & Watanabe, 2018), and that encourages the adoption of a new social role as a mother, especially for nulliparous women (Hennekam, 2016).

While McBride et al.'s (2003) model (hereafter referred to as the TM model) appears to explain pregnancy as a teachable moment, Olander et al. (2016) have suggested that the Capability-Opportunity-Motivation Behaviour model (COM-B; Michie, van Stralen, & West, 2011) provides a broader explanation of health behaviour change during pregnancy. The COM-B model has been posited as a ‘behaviour system’, that involves three essential conditions to generate behavioural change: capability (physical and psychological), opportunity (physical and social), and motivation (reflective and automatic). All conditions, except for reflective motivation, are thought to be necessary to generate a behaviour (Michie et al., 2011). Olander et al. (2016) argue that existing definitions of teachable moments rely mainly on motivation to explain behaviour change and that the COM-B model offers a greater understanding by moving beyond motivation and incorporating both an individual's capability and opportunity (also described as the ‘context’) to change their behaviour.

Whilst both the TM and COM-B models appear to offer relevant insight into the process of antenatal behaviour change, neither has been widely tested in a pregnant population, and a model of behaviour change specific to pregnancy does not exist. Pregnancy is a unique physiological event (Soma-Pillay et al., 2016) that requires women to make decisions about the life of another individual (her fetus), to engage with an ordered healthcare plan, and to manage societal expectations about their own behaviour. Unlike other teachable moments in acute health settings (e.g. hospitalisation, clinical appointments) (Meltzer et al., 2019; Son, 2015), pregnancy typically lasts up to forty weeks and is an evolving physiological process that requires women to make changes across multiple health behaviours. As such, it has been argued that throughout pregnancy individual events may create multiple individual teachable moments (e.g. feeling the baby move for the first time, attending key antenatal appointments) (Olander et al., 2016). It is therefore necessary to develop an understanding of behaviour change that is specific to the pregnancy experience, and for a behavioural model to be developed in the context in which it is intended to be applied.

In order to better understand pregnancy as a teachable moment for behaviour change, existing models applied to this context need to be examined to identify which psychological constructs are meaningful to the pregnancy experience, to establish which model might be best utilised in this context, and to identify where adaptations may be needed. Gaining this understanding will highlight elements of the models that are relevant, or irrelevant, within the context of pregnancy and provide insight that will contribute to the development of an enhanced pregnancy-specific model.

A previous systematic review and meta-synthesis identified factors influencing health behaviour change during pregnancy, specific to dietary behaviour, physical activity, smoking, and alcohol use (Rockliffe et al., 2021). This was an important first step in understanding pregnancy as a teachable moment. The current study aims to build upon this understanding by (1) assessing to what extent these factors are accounted for in the TM and COM-B models and (2) identifying which model is best used to understand behaviour change during pregnancy.

## Methods

Details of the systematic review and meta-synthesis have been reported in full elsewhere (Rockliffe et al., 2021). In brief, four bibliographic databases were searched (MEDLINE, PsycINFO, CINAHL-P, and MIDIRS) on 11/12/18 (and updated on 02/09/20) for studies providing qualitative data about women's experiences or perceptions of behaviour change specific to dietary behaviour, physical activity, smoking, and alcohol use, during an uncomplicated pregnancy. Hand searching and grey literature searches were also performed.

The search strategy retrieved 46,940 records. After removal of duplicates (*n *= 16,088) the titles and abstracts (*n *= 30,852) were screened by LR and DS, and full texts screened (*n *= 159) by LR and SP. Additional records were identified using alternative search methods (*n *= 36). Ninety-two studies were included in the review (see Rockliffe et al., 2021 for list of included studies). Study data were extracted using a data extraction tool, and study quality was assessed using a modified version of the Critical Appraisal Skills Programme qualitative appraisal checklist (CASP; Critical Appraisal Skills Programme, 2018).

Included studies were journal articles (*n* = 62, 67%), dissertations/theses (*n* = 29, 32%), and a research report (*n* = 1, 1%). These studies comprised 1,889 participants and were published between 1990 and 2020, most of which were conducted in the United Kingdom (*n* = 39, 42%). The majority of studies included women in the antenatal period only (*n* = 54, 59%), 28% (*n* = 26) included those in both the antenatal and postnatal period, and 13% (*n* = 12) included only postnatal participants. Where reported, 57% of studies (*n* = 52) included both nulliparous and multiparous women. Study characteristics are provided in the supplementary material of the original article.

Extracted data from the results sections of included studies were thematically synthesised (Thomas & Harden, 2008). Both author interpretations and participant quotes were coded line-by-line, using an inductive approach, before developing descriptive and higher-order themes. Papers containing references to dietary behaviour were analysed first, before using the thematic framework to guide the analysis of the papers containing references to physical activity, smoking, and alcohol use. Where additional codes were identified, these were incorporated into the thematic framework. Three overarching themes and nine sub-themes were generated from the data, which reflected factors present in the literature that influence women's antenatal health behaviour. These were entitled (1) A time to think about ‘me’, (2) Adopting the ‘good mother’ role, and (3) Beyond mother and baby. See Table 1 for details of the thematic framework. The themes and sub-themes apply to varying degrees across all four behaviours.

**Table 1.** Themes identified in thematic synthesis (Rockliffe et al., 2021).

Themes	Sub-themes	
1. A time to think about ‘me’	1.1.	A desire to self-indulge
	1.2.	A desire to retain ownership over body & behaviour
	1.3.	A desire for good health [mental & physical]
2. Adopting the ‘good mother’ role	2.1.	Driven by the health of the baby
	2.2.	Driven by roles & expectations
	2.3.	Driven by pre-pregnancy attitudes & behaviours
3. Beyond mother & baby	3.1.	Practical & environmental influences
	3.2.	Social influences
	3.3.	Knowledge, understanding, & advice

### Analytical approach

In the current study, a deductive approach was used to assess the extent to which the sub-themes identified in the thematic synthesis were accounted for by the TM model and COM-B model. The identified sub-themes were mapped to each of the models in turn. Sub-themes were mapped to the models, rather than the raw data from the systematic review, as the sub-themes summarise and reflect those data comprised within them. This mapping approach was unique to this study, although similar approaches have been reported elsewhere in the literature (e.g. Flannery et al., 2018; Gould, 2014; Rahman et al., 2021).

The mapping task was undertaken independently by LR and DS, then compared and contrasted to identify differences in the way the sub-themes had been mapped to the model constructs. Where differences existed, LR and DS discussed their interpretation and understanding of each of the sub-themes and/or constructs, before coming to a joint agreement as to the appropriate placement. In some instances, sub-themes were mapped to more than one construct. The agreed criteria for mapping the sub-themes to the models are presented in Tables 2 and 3. Mapping of the sub-themes was finalised following discussions with the wider research team.

**Table 2.** Criteria used to map sub-themes to the TM model constructs.

Model construct	Criteria
Risk perceptions or outcome expectations	The sub-theme should describe perceived risk or outcome expectancies related to the women's health or that of the pregnancy or baby. It can also describe any factors influencing a woman's perception of risk
Increased affective or emotional response	The sub-theme should describe an emotional state or response in relation to the pregnancy directly, or external pregnancy-related factors (e.g. social, environmental, practical)
Redefinition of self-concept or social role	The sub-theme should describe any factors impacting on a woman's sense of identity during her pregnancy

**Table 3.** Criteria used to map sub-themes to the COM-B model constructs.

Model construct	Criteria
Capability	*Physical*
	The sub-theme should describe factors influencing a woman's ability to physically participate in a health behaviour
	*Psychological*
	The sub-theme should describe factors influencing a woman's understanding, or ability to make decisions about her health behaviour
Opportunity	*Social*
	The sub-theme should describe any social or societal factors influencing health behaviour
	*Physical*
	The sub-theme should describe any external environmental factors influencing health behaviour
Motivation	*Automatic*
	The sub-theme should describe any reference to passive decision-making and behaviours driven by emotions or desires
	*Reflective*
	The sub-theme should describe behaviour driven by active decision-making, based on prior experience or reflection on past experience

## Results

In this section, each model and mapped sub-themes is presented in turn, describing how the sub-themes align with the particular model constructs. Illustrative quotes from the original analysis are presented within the text.

### Mapping sub-themes to the TM model constructs

Seven of the nine sub-themes from the thematic synthesis mapped to constructs of the TM model. ‘A desire for good health’ (1.3.), ‘Driven by the health of the baby’ (2.1.), and ‘Knowledge, understanding, and advice’ (3.3.) mapped to ‘risk perceptions or outcome expectancies’.

‘A desire to self-indulge’ (1.1.), ‘A desire to retain ownership over body and behaviour’ (1.2.), ‘A desire for good health’ (1.3.), ‘Driven by the health of the baby’ (2.1.), and ‘Driven by roles and expectations’ (2.2.) mapped to the ‘increased affective or emotional response’ construct.

‘Driven by roles and expectations’ (2.2.) and ‘Driven by pre-pregnancy attitudes and behaviours’ (2.3.) both mapped to the ‘redefinition of self-concept or social role’ construct.

The sub-themes ‘Practical and environmental influences’ (3.1.), and’ Social influences’ (3.2.), did not map to any of the model constructs. ‘A desire for good health’ (1.3.) mapped to several constructs, however the model did not account for women's experiences of physical symptoms captured within this sub-theme. This reflects a gap in the model where influences external to the woman and her pregnancy, or influences beyond her immediate control, such as physical symptoms, are not fully accounted for.

**Figure 1.**
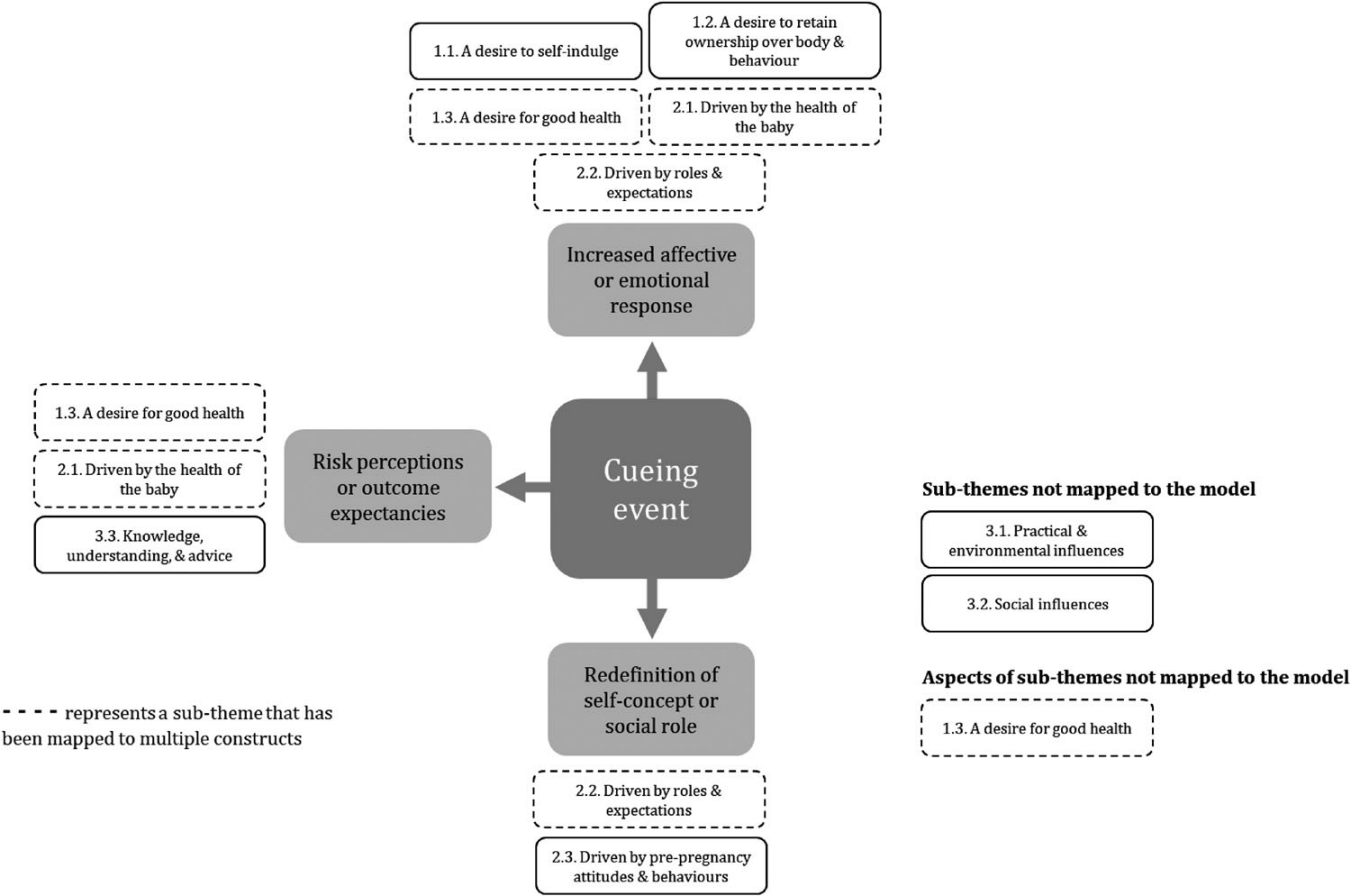
Mapping of sub-themes to the TM model constructs.

Figure 1 provides a visual representation of the way the sub-themes mapped to the constructs of the TM model.

#### Risk perceptions or outcome expectancies

Women's desire to ensure the health of their unborn baby (2.1. ‘Driven by the health of the baby’), as well as their own health (1.3. ‘A desire for good health’), acted to influence their health behaviour. Underlying this desire for good health were concerns about risks and negative outcomes associated with carrying out certain health behaviours. Women were keen to support the healthy development of their pregnancy, although the way in which they did this was often dependent on their understanding of the associated risks and the advice they received (3.3. ‘Knowledge, understanding, and advice’).

One of the women who had not sought any advice and did not receive any was worried about the health of the baby when exercising […] Lack of the right advice meant that the fears of exercising took over and she stopped all exercise as a result. (Trevorrow, 2016)

Women were sometimes driven to make healthy changes as they believed doing so would result in positive outcomes, such as increasing the chances of experiencing an uncomplicated pregnancy or birth. Some women also believed that eating healthily and maintaining physical activity would aid with their post-birth recovery, and positively impact their future health, which often motivated change.

For some reason I just had it in my head that if I stayed active during my pregnancy I would have an easier labour, and then I’d recover quicker afterwards. (Smith, 2017)

#### Increased affective or emotional response

Perceived risks to mother and baby (2.1. ‘Driven by the health of the baby’; 1.3. ‘A desire for good health’) elicited feelings of worry and concern, which in many cases encouraged healthy changes. However, modifying existing behaviours such as smoking or consuming alcohol, or engaging in physical activity, was also perceived to be risky by some, which could discourage change.

During this pregnancy, I have tried to reduced [sic] my smoking, I am trying if I can quit once and for all … just for the sake of my baby … I willn't [sic] want anything bad to happen. (Agberotimi, 2013)

More generally, women's mental state or emotional functioning influenced their motivation and decision-making in both positive and negative ways (1.3. ‘A desire for good health’). Whilst positive emotions and feeling mentally healthy could facilitate positive choices, negative emotions and unhelpful coping strategies could also act as barriers to change. In particular, feelings of judgment and shame had a bidirectional influence (2.2. ‘Driven by roles and expectations’).

[My friend] stopped smoking at work because she was like, “People are hating me down there! … I feel like I’m going to be lynched … Smokers are staring at me like I’m the devil!” (Murray, Small, & Burrage, 2014)

Some women experienced feelings of desire in relation to behaviours they experienced as being pleasurable or beneficial to their sense of well-being (1.1. ‘A desire to self-indulge’). This desire often acted to facilitate unhealthy behaviours such as eating increased quantities of food or continuing to smoke or consume alcohol. Conversely, it also encouraged some women to maintain physical activity. For some, the desire to self-indulge was at odds with their desire to do the best for the baby. This sometimes generated feelings of guilt, which could act as a barrier to smoking cessation (2.1. ‘Driven by the health of the baby’).

This suggests that for her total abstinence [from alcohol] is not necessary, and when desires are sufﬁciently strong, she can pay attention to them*.* (Atkinson, Shaw, & French, 2016)

Women's desire to retain control over their health behaviours (1.2. ‘A desire to retain ownership over body and behaviour’) acted as a barrier to change for some women, who were reported to resist advice and involvement from others and listen primarily to their own intuition, although this could also facilitate healthy choices. Additionally, some women were motivated to improve their behaviours in an attempt to assert control over gestational weight gain and their changing maternal body.

#### Redefinition of self-concept or social role

During pregnancy, women were keen to adopt what they perceived to be the role of a ‘good mother’. This sometimes involved modifying their health behaviour in an attempt to demonstrate they were putting their baby first and to comply with societal pressures and expectations (2.2. ‘Driven by roles and expectations’). However, some women were determined to disprove perceived stereotypes surrounding how pregnant women should behave, by continuing to exercise or consume some alcohol. Where women felt disconnected from their pregnancy and new identity as a mother, this could act as a barrier to smoking cessation.

… it was just a transition between who I was then, a young smoker and free, to being someone who was responsible for a child. So, once I stopped [smoking] I felt that that was my first responsibility for that child. (Ashwin, Marshall, & Standen, 2012)

Women's motivation to adopt the ‘good mother’ role was sometimes influenced (in both positive and negative ways) by their pre-pregnancy ‘self’, which was based on established attitudes and beliefs, behaviours, and skills (2.3. ‘Driven by pre-pregnancy attitudes and behaviours’). Fear of losing their pre-pregnancy identity acted as a barrier to change for some women. Prior experience of pregnancy or motherhood, or a lack thereof, also influenced women's decision-making.

I would argue that drinking alcohol, especially wine is seen as a significant social act, and a firm part of a woman's pre-pregnancy identity. Continuing to drink alcohol [during] pregnancy may therefore be a continuation of this expression of identity*.* (Ford, 2013)

### Mapping sub-themes to the COM-B model constructs

All nine sub-themes from the thematic synthesis mapped to the six constructs of the COM-B model. ‘A desire for good health’ (1.3.) and ‘Knowledge, understanding, and advice’ (3.3.) mapped to the ‘physical capability’ and ‘psychological capability’ constructs of the model, respectively.

‘A desire to self-indulge’ (1.1.), ‘A desire for good health’ (1.3.), ‘Driven by roles and expectations’ (2.2.), and ‘Driven by pre-pregnancy attitudes and behaviours’ (2.3.) mapped to ‘automatic motivation’, whilst ‘A desire to self-indulge’ (1.1.), ‘A desire to retain ownership over body and behaviour’ (1.2.), ‘A desire for good health’ (1.3.), and ‘Driven by the health of the baby’ (2.1.) mapped to ‘reflective motivation’.

‘Driven by roles and expectations’ (2.2.) and ‘Social influences’ (3.2.) mapped to ‘social opportunity’, and ‘Practical and environmental influences’ (3.1.) mapped to ‘physical opportunity’.

**Figure 2.**
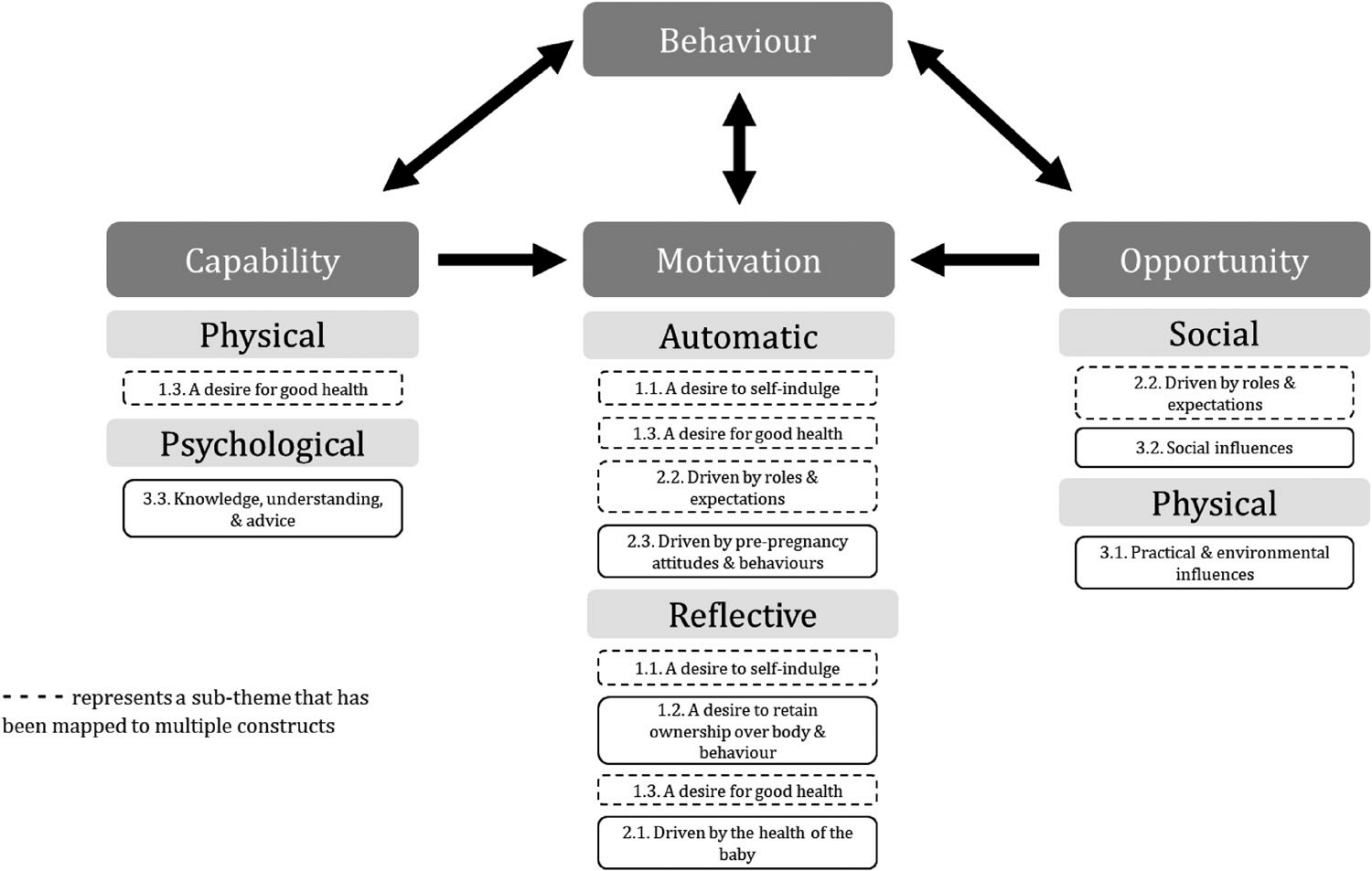
Mapping of sub-themes to the COM-B model constructs.

Figure 2 provides a visual representation of the way the sub-themes mapped to the constructs of the COM-B model.

#### Capability

##### Physical

Women's physical capability to undertake healthy behaviours was sometimes compromised by pregnancy symptoms (1.3. ‘A desire for good health’). For example, some women were unable to eat a healthy, balanced diet due to symptoms of sickness and nausea, changes in food preferences and/or cravings. Other symptoms such as bodily pain, fatigue, and/or nausea also restricted some women's ability to engage in physical activity.

I underestimated how tired I would be and how sick I’d feel … because of the nausea the last thing you want to do is exercise. (Cioffi et al., 2010)

##### Psychological

Women's psychological capability to make decisions about their health behaviour was affected by their levels of knowledge and understanding, and the advice they received (3.3. ‘Knowledge, understanding, and advice’). Those who had high levels of knowledge about the impact of their health behaviours on their pregnancy were better positioned to make healthy choices. Conversely, women who were less informed, who held misconceptions about the impact of their behaviours, or who did not have the skills necessary to make healthy changes, were less likely to do so. Support and advice received from health professionals also fed into decision-making, which could act as either a barrier or facilitator to change.

For those participants who gained excessive weight during their pregnancy, they voiced that if their midwife had informed them of this, they would have been more likely to make changes to their diet and levels of activity*.* (Smith, 2017)

#### Motivation

##### Automatic

Women were often keen to indulge themselves during pregnancy and engage in behaviours that they perceived to be pleasurable, such as eating, drinking alcohol, or smoking (1.1. ‘A desire to self-indulge’). This was driven by a sense that rules around food intake were relaxed during pregnancy, or because the behaviours were already established pre-pregnancy. In such cases, women appeared to be motivated by automatic impulses and desires, rather than conscious decision-making.

… relaxed social pressures of the slender ideal, accepting of increased food intake in pregnancy, when coupled with previous learned behaviours and habits gave rise to intemperance* … * (Tweheyo, 2016)

Furthermore, some women's mental and emotional states influenced their decision-making about their health (1.3. ‘A desire for good health’). Negative emotions (e.g. boredom, stress, frustration) sometimes drove unhealthy eating behaviour, and behaviours such as smoking and alcohol use were used as coping mechanisms by some women. These behaviours sometimes prompted feelings of shame and judgement, although this was also experienced in relation to physical activity. This could have a bidirectional influence on behaviour, in the sense that some women were motivated to make healthy changes, whilst others reported concealing their behaviour or increasing their smoking habits in response (2.2. ‘Driven by roles and expectations’). Conversely, positive emotions and attributes (e.g. having a positive attitude, increased levels of confidence and self-efficacy) sometimes facilitated change (1.3. ‘A desire for good health’).

One pregnant female who was in the process of quitting was motivated by guilt and stigma: ‘‘[…] It makes me ashamed and looks bad as no one likes smokers these days.’’ (Bull et al., 2007)

Automatic processes relating to established pre-pregnancy habits and routines also influenced health behaviour (2.3. ‘Driven by pre-pregnancy attitudes and behaviours’). Women with existing un/healthy behaviours often maintained these during pregnancy. Similarly, established beliefs and attitudes towards the idea of making behavioural changes during pregnancy also influenced decision-making. Prior experience of pregnancy, or previous pregnancy difficulties, fed into behavioural decisions for some women.

… growing up you always get these kind of, you know, like ‘this is the way you should do it, this is the way you shouldn't do it’ and it all just kicks in at the same time […] I felt like it was just all my instincts just pushing us (Laing, 2015)

##### Reflective

Some women engaged in a reflective decision-making process based on a desire to retain control and ownership over their health behaviours (1.2. ‘A desire to retain ownership over body and behaviour’). This meant responding intuitively to what they believed their body or baby needed, which for some women meant avoiding making healthy changes. Women also reported a reflective motivation to make healthy changes to their eating behaviour and physical activity levels, based on a desire to exert control over their changing maternal bodies.

Physical activity was a strategy utilised by each woman in this study to control the way they felt about their bodies, and to manage their feelings about the changes their bodies were subject to*.* (Dormer, 2019)

In making decisions about their health behaviour, it was evident that reflecting on anticipated outcomes in relation to the health of the baby (2.1. ‘Driven by the health of the baby’) and on their own health (1.3. ‘A desire for good health’) were important influences. Women reported concerns about experiencing pregnancy or birth complications and were keen to avoid causing harm to their unborn baby as a consequence of engaging in poor health behaviours. However, some women reported concerns that ceasing behaviours such as smoking and drinking, might also have negative repercussions. Feelings of guilt were reported by some women when their behaviours did not align with their good intentions, which sometimes exacerbated poor health behaviours.

… I don't want my baby, you know, affected by it, it's just I want it to, you know, have a proper start, if it came out with any defects or anything through alcohol and that was my fault, I’d never forgive myself. (Laing, 2015)

As a result of maintaining or initiating healthy behaviours, some women experienced improvements in their mental health and well-being. Reflecting on this further motivated these women to maintain their healthy behaviours, and some viewed prioritising these activities (i.e. physical activity) as an indulgence (1.1. ‘A desire to self-indulge’). Reflecting on the potential benefits to postnatal health also influenced some women's decision-making.

#### Opportunity

##### Social

Social opportunity impacted on women's motivation to make changes to their behaviour, to varying degrees (3.2. ‘Social influences’). Levels of support from those around them influenced women's behaviour, as did social norms (e.g. where smoking and drinking were considered the norm) and the behaviour of others. Health behaviours that are by nature social activities, such as drinking alcohol or smoking, were difficult to modify, especially where they were important features of social relationships. However, the social nature of physical activity could act as a facilitator.

If friends or acquaintances who were currently or recently pregnant ate more unhealthy food during pregnancy it was easy to be influenced to also eat this. (Andersson & Ernstsson, 2017)

Societal roles and expectations (2.2. ‘Driven by roles and expectations’), which is another form of social opportunity, also influenced decision-making. Some women were motivated to change their behaviours due to a desire to adhere to the ‘good mother’ role, and/or in response to societal pressure and expectations. Feelings of judgement and stigma surrounding certain behaviours (as previously discussed) could act as a barrier or a facilitator to behavioural change.

Social norms, dictating that expectant mothers should avoid alcohol, were cited as the main reasons why women stopped drinking. (Schölin et al., 2017)

##### Physical

The physical environment and related external influences impacted on women's opportunity to make changes to their behaviour (3.1. ‘Practical and environmental influences’). Environments that promoted un/healthy behaviours or increased availability of un/healthy choices influenced some women's decision-making. Practical factors such as lack of familiarity with the physical environment, the weather, and accessibility of appropriate exercise classes/spaces also influenced motivation to be physically active. Time constraints, competing priorities, financial considerations, and availability and/or accessibility of support services influenced some women's decision-making and motivation, as did socio-economic disadvantage.

I have spent sometimes £15 on one meal that is going to be really healthy and I could just go buy a pizza for £2. (Chana & Haith-Cooper, 2019)

## Discussion

The purpose of this study was to understand to what extent factors identified as influencing antenatal health behaviour were accounted for by the TM model and COM-B models of behaviour change, and to establish which model is best used to understand behaviour change during pregnancy.

Results of the theoretical mapping exercise revealed that all sub-themes identified in the previous review mapped to the COM-B model, indicating that all influencing factors play a role in directing behaviour during pregnancy. However, the TM model failed to account for several sub-themes (1.3. ‘A desire for good health’; 3.1. ‘Practical and environmental influences’; 3.2. ‘Social influences’) which mapped to the ‘physical capability’, ‘physical opportunity’, and ‘social opportunity’ constructs of the COM-B model, respectively.

The three sub-themes not accounted for by the TM model reflect non-psychological factors that influence behaviour, such as those in the physical or social environment, or the experience of pregnancy symptoms. The existing TM model constructs capture women's internal cognitive processes relating to risk, emotions, and identity, but fail to account for any factors beyond these internal psychological processes or those presented in the external environment. Accordingly, the unmapped sub-themes that mapped to the ‘opportunity’ construct of the COM-B model (also described as the ‘context’) are defined as factors that ‘lie outside the individual’ (Michie et al., 2011). Numerous studies have highlighted the important role that social, and practical or environmental factors play in women's decision-making around their health during pregnancy (Harrison et al., 2018; Omidvar et al., 2018; O’Brien et al., 2017). Furthermore, physical pregnancy symptoms are commonly cited as a barrier to behaviour change, particularly in relation to dietary behaviour and physical activity (Foxcroft et al., 2011; Harrison et al., 2018; Swift et al., 2017). As such, and as these factors were also captured in the COM-B model, the addition of a construct reflecting non-psychological factors may enhance the overall utility of the TM model within the context of pregnancy.

Physical pregnancy symptoms are often transient for women, frequently occurring most severely during the first trimester (Smith et al., 2000). The study findings highlight the need to consider factors such as these, that are changeable, as it has implications for the timing of interventions if certain stages of pregnancy present more barriers to change, than others. It is therefore important to reflect upon whether other factors mapped to the models, or the model constructs themselves, change or whether they remain stable throughout pregnancy. For example, a women's psychological capability may increase over time as she acquires new knowledge, or risk perceptions may reduce. Whilst this wasn't explored explicitly in the original review, it has previously been suggested that pregnancy may provide several individual teachable moments for behaviour change, prompted by significant events (e.g. receiving confirmation of the pregnancy, feeling the baby kick for the first time) (Olander et al., 2016). Neither the TM model nor COM-B model offer a heuristic that accounts for potential changes in the salience of factors influencing health behaviour, strengthening the argument for the development of a pregnancy-specific model. Further qualitative inquiry is necessary to explore the different factors influencing health behaviour throughout pregnancy, to identify whether certain stages afford more powerful teachable moments than others.

Our findings identified some similarities and overlap between the constructs of the two models. For example, two of the sub-themes mapped to the ‘automatic motivation’ construct of the COM-B model also mapped to the ‘increased affective or emotional response’ construct of the TM model. This makes sense as the ‘automatic motivation’ construct is defined as involving emotions and impulses (Michie et al., 2011). Similarly, the sub-theme mapped to the ‘psychological capability’ construct of the COM-B model also mapped to the ‘risk perceptions or outcome expectancies’ construct of the TM model. This also makes sense, as risk perception relies on psychological processes to assess the probability of negative outcomes, based on knowledge acquisition (Johnson, 1993). However, this highlights the different ways in which the model constructs have been conceptualised, which is the key difference between the two models.

The COM-B model is a general behaviour system designed to incorporate various contexts (Michie et al., 2011), which may explain why all sub-themes mapped to the respective constructs. The model constructs are intentionally broad and encompassing, owing to the nature of its design (Michie et al., 2011), however this has previously been highlighted as a limitation, as it restricts the ability to test and falsify the model in any given context (Ogden, 2016). Whilst Olander et al. (2016) suggest that the COM-B model may provide an enhanced analysis of behaviour in pregnancy, by considering factors beyond motivation alone, the model's lack of specificity limits its ability to be fully applied to this context. Conversely, the TM model has been designed to specifically understand the way in which health events may act as teachable moments for behaviour change (McBride et al., 2003). Pregnancy is often conceptualised as a teachable moment, or series of teachable moments (Olander et al., 2016; Phelan, 2010), as it is a health event that prompts women to consider associated health risks, that may provoke a strong emotional response, and that may cause women to reflect upon their own identity as a mother (Phelan, 2010). As such, the TM model provides a level of conceptual understanding than goes beyond what the COM-B model can offer. This is evident from the way in which the sub-themes mapped to the constructs; For example, sub-themes from each of the three overarching themes mapped to the ‘risk perceptions or outcome expectancies’ construct of the model, highlighting the way in which risk is interwoven throughout women's experience of pregnancy. Similarly, all sub-themes within the higher order theme ‘A time to think about “me”’ mapped to the ‘increased affective or emotional response’ construct, emphasising the emotional impact of pregnancy in relation to women's own needs and desires.

It is interesting to note that some of the TM model constructs did not explain various pregnancy behaviours in the way in which the model originally posits. McBride et al. (2003) state that events eliciting a strong emotional or affective response, whether positive or negative, may increase the likelihood of a teachable moment. However, for some women elicitation of strong emotions, such as shame or judgment, led them to reduce their physical activity levels or increase their smoking behaviour. It may be that this is pregnancy-specific and an additional limitation of applying this model to this health context. Conversely, the COM-B model does not suggest a directional relationship between model constructs and behaviour. It may be beneficial to explore this further in future work, to better understand how the same factors can influence behaviour in opposing ways.

An additional observation is that for both models more sub-themes mapped to certain constructs than others. For example, within the TM model more sub-themes mapped to ‘increased affective or emotional response’ than any other constructs. Similarly, within the COM-B model the most sub-themes mapped to the ‘motivation’ constructs. It is possible that this indicates these constructs play a bigger role in directing behaviour than the others. However, further research would be required to assess how much variance each of the model constructs explain of a target behaviour, using a quantitative approach.

### Strengths and limitations

This study has generated novel findings that have highlighted the relative merits and limitations of existing models of teachable moments when applied within the context of pregnancy. However, there are some limitations of this study that should be acknowledged. Firstly, the sub-themes used in the mapping exercise were generated from an analysis focused on pregnancy-related behaviours only (i.e. behaviours that also occur outside of pregnancy, such as dietary behaviour, smoking etc) rather than pregnancy-specific behaviours (i.e. those that occur during pregnancy only, that are initiated during this period, such as recommended supplementation) (Olander, Smith, & Darwin, 2018). It is therefore possible that the models might be better suited to understanding certain behaviours over others. Furthermore, it is important to consider that within the behaviours included in the analysis, the models, or certain model constructs, might be more relevant to particular behaviours. For example, the TM model was developed in the context of smoking cessation and is therefore frequently used to understand this behaviour, above others (Baron et al., 2017; McBride et al., 2003; McBride, Blocklin, Lipkus, Klein, & Brandon 2017). Furthermore, the ability of the COM-B model to explain behaviour has been suggested to vary across behavioural contexts; in particular, it has been found to better explain physical activity than it has eating behaviour (Willmott, Pang, & Rundle-Thiele, 2021). With this in mind, it may be beneficial to further explore differences between behaviours by conducting individual mapping exercises. Whilst the sub-themes used in the analysis reflected factors that influenced all four behaviours, there was some degree of variability in the extent to which they applied to each. To capture any such nuance, it may therefore be advantageous to map the raw data from the original analysis to the model constructs, rather than the sub-themes.

Secondly, the mapping exercise relied on judgments as to which constructs the sub-themes were mapped to. Whilst two of the authors completed the mapping task independently to enhance reliability, there is still an element of subjectivity that will have guided the task. Furthermore, it is important to highlight that limitations of the original review also have implications for interpreting the findings of this study. In particular, it is likely that individual variability (i.e. parity), cultural differences, and differences in healthcare advice provided in different countries will have influenced women's health behaviour, both implicitly and explicitly.

Whilst the findings of the mapping exercise provide us with an improved understanding about the utility of existing models, we are unable to draw firm conclusions without empirical data to support our suppositions. It will therefore be beneficial to further explore these findings and test the models using a more structured quantitative approach.

### Implications for theory development

The findings from this study provide valuable insight that will contribute towards the development of an enhanced pregnancy-specific model. Whilst the COM-B model provides an overall ‘good-fit’ in terms of influencing factors mapped to the model, it lacks a conceptual understanding relevant to the pregnancy experience. Conversely, the TM model provides a level of conceptualisation that appears relevant to the pregnancy experience but fails to account for non-psychological factors. Furthermore, the TM model does not appear to explain the bidirectionality of some pregnancy behaviours (i.e. the way in which the same influencing factor may encourage both healthy and unhealthy behaviours), and neither model accounts for changes in the salience of influencing factors over time. Longitudinal research is therefore necessary to better understand how the models operate at different stages throughout pregnancy; measuring women's health behaviour (e.g. eating behaviour) and the model constructs at multiple gestational stages would provide an enhanced understanding of how the model constructs change throughout pregnancy and to what extent they explain behaviour.

It will be important to consider all of these elements during theory development and to build upon the knowledge we have generated. By combining the existing TM model constructs with a construct that reflects non-psychological factors, including physical symptoms, social influences, and practical and environmental influences, we may be able to develop an enhanced model that is more relevant and meaningful to the pregnancy context. It will also be valuable to consider the evolving nature of pregnancy and the relevance of model constructs at different points throughout pregnancy.

## Conclusions

Both the COM-B model and TM model have limitations when applied within the context of pregnancy. However, each model contains elements which will be crucial in the development of a pregnancy-specific model of behaviour change. Going forward, it is necessary to develop a model that adequately conceptualises women's pregnancy experiences and accounts for non-psychological influencing factors, in additional to internal cognitive processes. Combining aspects of each model will be key to developing an enhanced model that is appropriate and effective in supporting women to improve their health behaviour during pregnancy.
